# The imbalance of PGD2-DPs pathway is involved in the type 2 diabetes brain injury by regulating autophagy

**DOI:** 10.7150/ijbs.60149

**Published:** 2021-09-21

**Authors:** Yang Yang, Pu Xiang, Qi Chen, Ying Luo, Hong Wang, Huan Li, Lu Yang, Congli Hu, Jiahua Zhang, Yuke Li, Hui Xia, Zhihao Chen, Junqing Yang

**Affiliations:** 1 Department of Pharmacology, Chongqing Medical University, the Key Laboratory of Biochemistry and Molecular Pharmacology, Chongqing 400016, China.; 2 Department of Pharmacology, Chongqing Health Center for Women and Children Chongqing 400016, China.; 3 Department of pharmacy,Dianjiang People's Hospital of Chongqing, Dianjiang, Chongqing 408300, China.; 4 Pharmacy department of GuiZhou Provincial People,s Hospital, Guiyang 550000, China.

**Keywords:** type 2 diabetes, brain injury, PGD2, PKA, autophagy

## Abstract

Prostaglandin D2 (PGD2) is the most abundant prostaglandin in the brain, but its involvement in brain damage caused by type 2 diabetes (T2D) has not been reported. In the present study, we found that increased PGD2 content is related to the inhibition of autophagy, which aggravates brain damage in T2D, and may be involved in the imbalanced expression of the corresponding PGD2 receptors DP1 and DP2. We demonstrated that DP2 inhibited autophagy and promotedT2D-induced brain damage by activating the PI3K/AKT/mTOR pathway, whereas DP1enhanced autophagy and amelioratedT2D brain damage by activating the cAMP/PKA pathway. In a T2D rat model, DP1 expression was decreased, and DP2 expression was increased; therefore, the imbalance in PGD2-DPs may be involved in T2D brain damage through the regulation of autophagy. However, there have been no reports on whether PKA can directly inhibit mTOR. The PKA catalytic subunit (PKA-C) has three subtypes (α, β and γ), and γ is not expressed in the brain. Subsequently, we suggested that PKA could directly interact with mTOR through PKA-C(α) and PKA-C(β). Our results suggest that the imbalance in PGD2-DPs is related to changes in autophagy levels in T2D brain damage, and PGD2 is involved in T2D brain damage by promoting autophagy via DP1-PKA/mTOR and inhibiting autophagy via DP2-PI3K/AKT/mTOR.

## Introduction

Diabetes mellitus is a common metabolic disorder. The prevalence of type 2 diabetes (T2D) is increasing as lifestyles change. T2D has become the main cause of disability and death in recent years 1 and was responsible for 1.5 million deaths in 2012. One of the most concerning problems of T2D is long-term neurological complications 2. As many as 60% to 70% of diabetic patients develop neuropathy 3, and T2D patients have a greater risk of developing brain atrophy and cognitive impairments 4. Cognitive deficits significantly affect quality of life and greatly increase the economic burden of diabetic patients 3. Although many factors involved in T2D-induced brain injury have been identified, the mechanism is still unclear.

Autophagy plays an important role in stabilizing the function of the central nervous system 5. The decrease in autophagy caused by the chronic deterioration of the autophagic lysosomal system of nerve cells might be one of the important pathophysiological mechanisms of AD 6, and the activation of autophagy reduced Alzheimer's disease-like pathology and cognitive decline in a murine model 7. Some studies 8 have indicated a decrease in LC3BII and increase in p62 in autism-induced hippocampal injury. Autophagy can be regulated by hyperglycaemia in diabetes, and dysfunctional autophagy is responsible for glucotoxicity 9, 10. However, how autophagy is regulated in T2D is not clear.

Cyclooxygenase (COX) is a rate-limiting enzyme involved in the synthesis of prostaglandins and in the regulation of the functions of the central nervous system (CNS) 11. Prostaglandin D2 (PGD2) is the most abundant prostaglandin in the brain and has been shown to be involved in many processes, including the regulation of sleep 12. Epidemiological investigations have shown that sleep disorders are common in T2D patients, and sleep disorders also promote brain damage caused by T2D 13, 14. Our previous results indicated that decreased autophagy may be related to PGD2 15. However, the mechanism of autophagy inhibition by PGD2 is unclear. PGD2 may participate in the regulation of autophagy through its receptors. PGD2 has two corresponding receptors, DP1 and DP2. However, DP1 and DP2 have not been reported in T2D brain injury, and it is unclear whether PGD2-DPs are involved in the regulation of autophagy. Activation of protein kinase A(PKA) occurs downstream of DP1, but there is no relevant report on whether PKA is directly involved in mTOR regulation.PKA plays various regulatory roles, mainly through its catalytic subunits (PKA-C subtypes, α, β, and γ), but whether they are all involved in mTOR regulation has not been studied.

Therefore, the present study was designed to investigate the effects of PGD2-DPs on brain injury and explore the mechanisms that may influence autophagy in T2D. These results may help clarify the mechanism of brain damage caused by T2D and aid the discovery of new therapeutic targets for T2D-induced brain damage.

## Materials and Methods

### Animals

Sprague-Dawley (SD) rats were housed in the barrier housing facility, and it has in keeping with national standard “Laboratory Animal-Requirements of Environment and Housing Facilities”. The care of laboratory animal and the animal experimental operation have conforming to “Chongqing Administration Rule of Laboratory Animal”. The experimental procedures were approved by the animal laboratory administrative center and the institutional ethics committee of Chongqing Medical University (License number: SYXK YU 2012-0001) and also in accordance with the National Institutes of Health guidelines. The rats were kept in controlled conditions of temperature (24 ± 2 °C), relative humidity (60 ± 10%) and 12/12 h light/dark cycle (light from 08:00 am to 08:00 pm).

The modeling method is based on our published article 15-17. To establish the rat model of T2D, 20 male rats (80-100 g, 4-week old) were fed high fat diet (HFD) (20% sugar, 10% lard, 10% egg yolk and 60% basal feed) after a week of normal diet. After 4 weeks, rats were injected once with low-dose streptozotocin (Solarbio, China) (STZ, 30 mg/kg i.p) to induce partial insulin deficiency, and then continuously fed HFD for 4 weeks after injection of STZ.10 male rats were lived after the completion of modeling. After the completion of modeling, 10 male rats were reared for another 12 weeks as a model group. There were 9 rats remaining in each group when the administration was completed. The rats of control group were fed normal diet.

### Morris water maze test

Morris water maze was used to evaluate spatial learning and memory function of rat in each group. Rats were given four trials per day for four consecutive days. A different entry site was used for each daily session. During each trial, the rats were introduced into the water where a hidden platform was submerged under the water. If rats failed to reach the platform within 90 sec, they were gently guided to it and allowed to remain for 10 sec on top of the platform. On the 5th day, following the last day of training, rats were introduced into the pool from the entry site where the last training was performed in order to assess retention of the platform location. During this probe trial, the platform was removed from the maze. The latency for rat to find the hidden platform and the number of times to cross the platform were recorded, with a maximum of 90 sec.

### Histopathological and Tunel observation

After the Morris water maze test, 3 rats from each group were perfused with heparinized saline (30 ml) to remove blood from the vasculature, and then with 4% paraformaldehyde in phosphate buffered saline (50 ml). The whole brain was then removed and stored in the same fixative. After paraffin embedding, 5-μm sections were obtained and stained with hematoxylin-eosin (H & E). Morphologic changes of hippocampus and cortical neurons were examined using light microscopy. Cells with a distinct nucleus and nucleolus were regarded as intact neurons. Tunel experiment is carried out according to the experimental procedure of the kit.

### Biochemical assays

A plasma level of insulin was measured by ELISA kits (*YuanYe, PRC*) according to the manufacturer's recommendation. The levels of Triglycerides (TG), Total Cholesterol (T-CHO) and Low-Density Lipoprotein Cholesterol (LDL-C) in bloodsamples were measured by commercial assay kits (*JianCheng, PRC*) according to the manufacturer's directions.

### Cell culture and treatments

Immortalized murine hippocampal HT22 cell lines were obtained from BNCC, China. HT22 cells were cultured in DME/F12 medium (Hyclone, United States) supplemented with 10% fetal bovine serum (FBS; Hyclone, United States) and 1% 100× penicillin-streptomycin (Gibco, United States) at 37 ◦ C and 5% CO 2.To establish the cell model, HT22 cells were cultured in high-glucose (HG, glucose reached 75 mM) medium for 36 h 18. The cells were divided into the control group, high-glucose overloaded group (HG group, the glucose reached 13.5 mg/mL in medium), HG+BW245C (DP1 agonist, 10^-5^ M, CAYMAN, USA) group, HG+BWA868C(DP1 antagonist, 10^-5^ M, CAYMAN, USA) group, HG+DK-PGD2 (DP2 agonist, 10^-5^ M, CAYMAN, USA) group, HG+CAY10471 (DP1 antagonist, 10^-5^ M, CAYMAN, USA) group, HG+DK-PGD2+Rapamycin (10^-11^ M, SELLECK, USA) group, HG+CAY10471+Wortmannin (10^-9^ M, SELLECK, USA) group.

### Enzyme-linked immunosorbent assay (ELISA)

After treatment, the content of PGD2 in rats cortex and hippocampus were detected with ELISA kits (Meibiao, Jiangsu, China). After treatment, the content of PGD2 and camp in HT22 cells were detected with ELISA kits (Meibiao, Jiangsu, China).

### MTT Assay

HT22 cells were cultured in the 96-well plates at 10×10^4^ cells/ml and subjected to HG and intervention for 36h. After the intervention, MTT (20 µL, 5 mg/ml; Sigma, United States, M2128) was added per well. After 2h of incubation, the medium was removed, and 150 µL DMSO was added to solubilize the purple formazan. Then, the plate shook slowly on the horizontal shaking table free from light for 10 min at room temperature. Finally, optical density (OD) was detected at 490 nm using a microplate reader (BioTek, United States; Lobner, 2000).

### LDH Leakage Rate Detection

Cell death was evaluated using the lactate LDH Assay Kit (Beyotime, China, C0017). HT22 cells were cultured in 96-well plates at 5 × 10^4^ cells/ml. After HG and intervention treatment for 36h, the LDH leakage rate in the cell culture supernatant was measured according to the manufacturer's instruction.

### Flow Cytometry Analysis

HT22 cells were seeded in six-well plates at 8 × 10^4^ cells/mL and exposed to HG and intervention for 36h. Afte the incubation period, cells were trypsinized without EDTA, collected and suspended in 1 ml PBS. Apoptosis was determined by flow cytometry using the Annexin V-FITC/propidium iodide (annexin V/PI) apoptosis detection kits according to the manufacturer's protocol. Three separate experiments were performed.

### Transmission Electron Microscopy Observation

The numbers of autolysosome were confirmed by transmission electron microscopy (TEM) examination. After treatment, HT22 cells were collected in glutaraldehyde at 4 °C.

### Co-Immunoprecipitation

IP lysates buffer were added to cells and tissues. 1.0 μg IgG and 20 μL A/G-beads were added to the cell lysis or tissue lysis, incubate at 4 °C for 1 h. The lysates were clarified by centrifugation at 2000 g for 5 min at 4 °C, and take the supernatant. The 10 μL mTOR antibodies were added to the supernatant. Then incubate overnight at 4 °C. Add 20 μL of A/G-beads and incubate for 2 h at 4 °C. Centrifuge at 1000 g for 5 min at 4 °C, remove the supernatant, and collect the immunoprecipitated complex. The immunoprecipitated complex was washed 4 times with 1 ml of ice-cold IP lysate, centrifuged at 1000 g for 5 min at 4 ° C each time, and the supernatant was carefully discarded for each wash. After the last wash, carefully discard the supernatant, add 40 μL of 1×SDS thioglycol-containing loading buffer, boiled for 10 min. At last, take 20 μL of supernatant sample for WB detection.

### The lentivirus infection

The lentivirus was bought from company (*CyagenBiosciences, Guangzhou, China*). When the HT22 cells were attached, the culture medium containing lentivirus and polybrene (5 ug/ml) was added it for 8 h. After 8 hours, the medium containing virus and polybrene was removed, and then a new virus-free and polybrene-free medium was added it. By observation, it was found that over 80 to 90% cells had fluorescence expression, which proved that lentivirus infection was successful and could be used in experiments (it can be screened with antibiotics).

### Western Blot Analysis

After treatment, HT22 cells were washed in ice-cold PBS and lysed by RIPA lysis buffer containing phosphatase and protease inhibitors. The samples were collected and centrifuged at 12,000 × g and 4 °C for 15 min, after incubated for 20 min on ice. The supernatant was collected and the total protein concentrations were measured with a BCA protein assay kit (Beyotime, China, P0010S). Loading buffer (Beyotime, China, P0015L) was added into the remaining supernatant and boiled at 100 °C for 15 min. Ultimately, samples were stored at -20 °C for further research. Fifty mg of rat cortex and hippocampus (n=4) were added to 0.5 ml of tissue lysate solution for protein extraction and centrifugation at 12,000 × g for 10 min at 4 °C, and the supernatant was used for detection of protein concentrations with a BCA protein assay kit *(Beyotime, China)*. A 10 μL sample of protein was separated by sodium dodecyl sulphate polyacrylamide gel electrophoresis (SDS-PAGE) and transferred to PVDF membranes (*Millipore, USA*). The membranes were blocked with 5% BSA for 1 h at room temperature and then probed with specific primary antibodies, including DP1 (1:1000; *Abcam, UK*), DP2 (1:500; *SANTA, USA*), LC3BII (1:1000; *Abcam, UK*), p62 (1:1000; *Abcam, UK*), AKT (1:1000; *Abcam, UK*), p-AKT (1:500; *SANTA, USA*), mTOR (1:1000; *Abcam, UK*), p-mTOR (1:1000; *Abcam, UK*), PKA-C (α/β) (1:500; *SANTA, USA*) and β-actin (1:4000; *Proteintech, USA*) overnight at 4 °C. The membranes were washed three times in TBST and incubated with HRP-conjugated secondary antibodies at room temperature for one hour. Following four washes in TBST, protein signals were visualized by ECL* (Bio-Rad, USA)*.

### Statistical Analysis

Data are presented as mean±standard deviation (SD). Statistical analysis was carried out using SPSS statistics software (Version 20.0) and data were analyzed by performing one-way analysis of variance (ANOVA) followed by post hoc Tukey's test (≥ three groups). Two-group variances were compared using Student's t test. P-value less than 0.05 were considered statistically significant.

## Results

### Changes in blood glucose, LDL-C, T-CHO, TG, body weight and insulin in T2D rats

**Figure 1:**
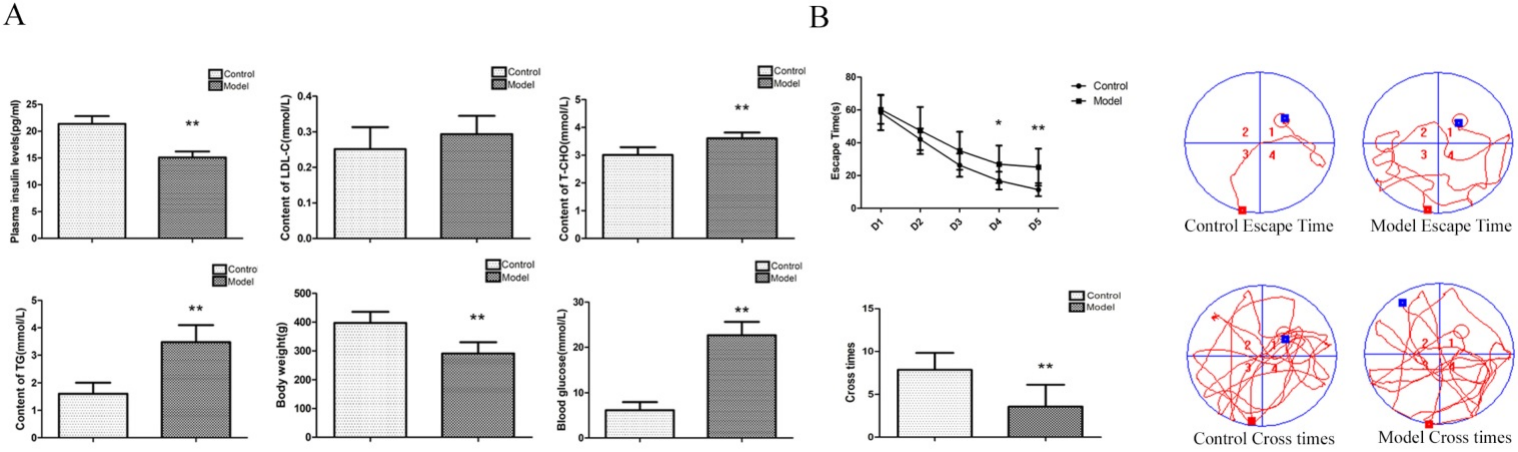
** Changes inbiochemical indicators andspatial learning and memory, neuronal pathomorphology, PGD2 content, and related protein in T2D rat. (A)** Biochemical indicators; **(B)** Morris Water Maze. Rats in the model group showed a significant increase of blood glucose, T-CHO and TG, a significant decrease of body weight and plasma insulin, there is no change of LDL-C; a significant increase of escape latency and a significant decrease of the number of platform cross times. Data are expressed as mean ± SD of nine individual rats in each group.^*^P<0.05 and ^**^P<0.01 compared with control group, respectively.

To determine whether our model was an effective T2DM model, we measured various biochemical indicators. Compared with the control group, the contents of blood glucose, T-CHO and TG were significantly increased; the body weight and the content of insulin were significantly decreased; and the content of LDL-C was not significantly changed in the model group (Figure 1A). The above indexes were combined with our previously published articles 15-17 about T2D modelling by this method. We successfully established a T2D model.

### Changes in spatial learning and memory abilities in T2D rats

To determine whether rats had neurological impairment, we used the Morris water maze to test their learning and memory abilities. The Morris water maze results showed that the rats in all groups exhibited a rapid reduction in their escape latencies to find the platform over the 4 training days. Compared with those in the control group, rats in the model group showed significantly prolonged escape latency and a decrease in the number of platform crossings on the 4^th^ and 5^th^ days (Figure 1B). These results indicated that T2Drats had learning and memory defects.

### Changes in neuronal pathomorphology inT2D rats

**Figure 2:**
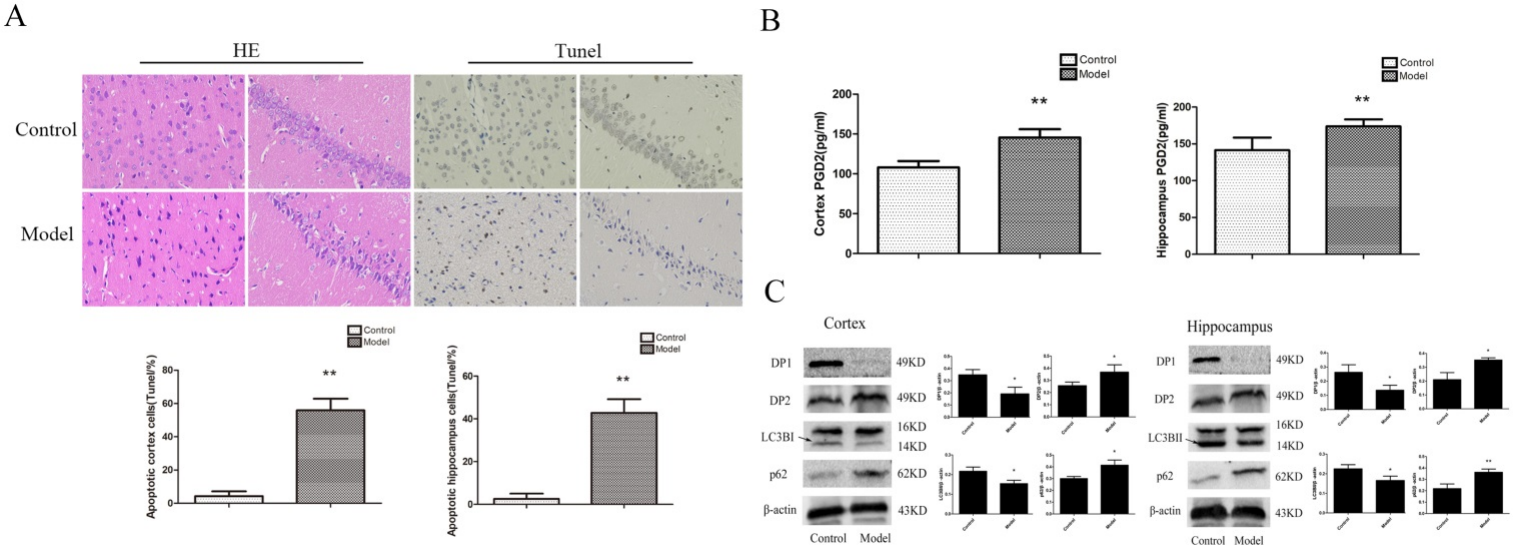
** Change inneuronal pathomorphology, PGD2 content, and related protein in T2D rats cortex and hippocampus. (A)** HE and Tunel (400×); **(B)** PGD2 content; **(C)** DP1, DP2, LC3BII and p62 expression. The number of apoptosis cells were significantly increased; the content of PGD2 was significantly increased; the DP1 andLC3BII expression were significantly decreased, the DP2 and p62 expression were significantly increased. Data are expressed as mean ± SD of nine individual rats in each group.^*^P<0.05 and ^**^P<0.01 compared with control group, respectively.

To determine whether the rat brain had pathological changes, we used HE and Tunel to test the changes in neuronalpathomorphology in the hippocampus and cortex. In the control group, the morphological neuronal structure of the hippocampus and cortex was intact and clear. Compared with the control group, the neurons in the model group showed remarkable karyopyknosis (Figure 2A). Tunel experiment showed that the numbers of apoptosis cells were significantly increased in the model cortex and hippocampus. These results indicated that T2D rats' neuronal damage was significantly increased.

### Changes inPGD2, DP1, DP2, LC3BII, and p62 expression in the hippocampus and cortex of T2D rats

To determine whether the change in PGD2-DPs and autophagy induced T2DM brain injury, we used ELISA and WB analyses to detect PGD2 and related protein expression in the rat cortex and hippocampus. LC3BII reflected the intensity of autophagy, while p62 reflected a decrease in degradation activity. Compared with the control group, the content of PGD2 was significantly increased, the expression of DP2 and p62 was significantly increased, and the expression ofDP1 and LC3BII was significantly decreased in the model group (Figure 2B, C). The above findings suggested that the imbalance of PGD2-DPs may be closely related to the decrease in autophagy levels in the hippocampus and cortex of T2D rats.

### The effect of HG on HT22 cells

**Figure 3:**
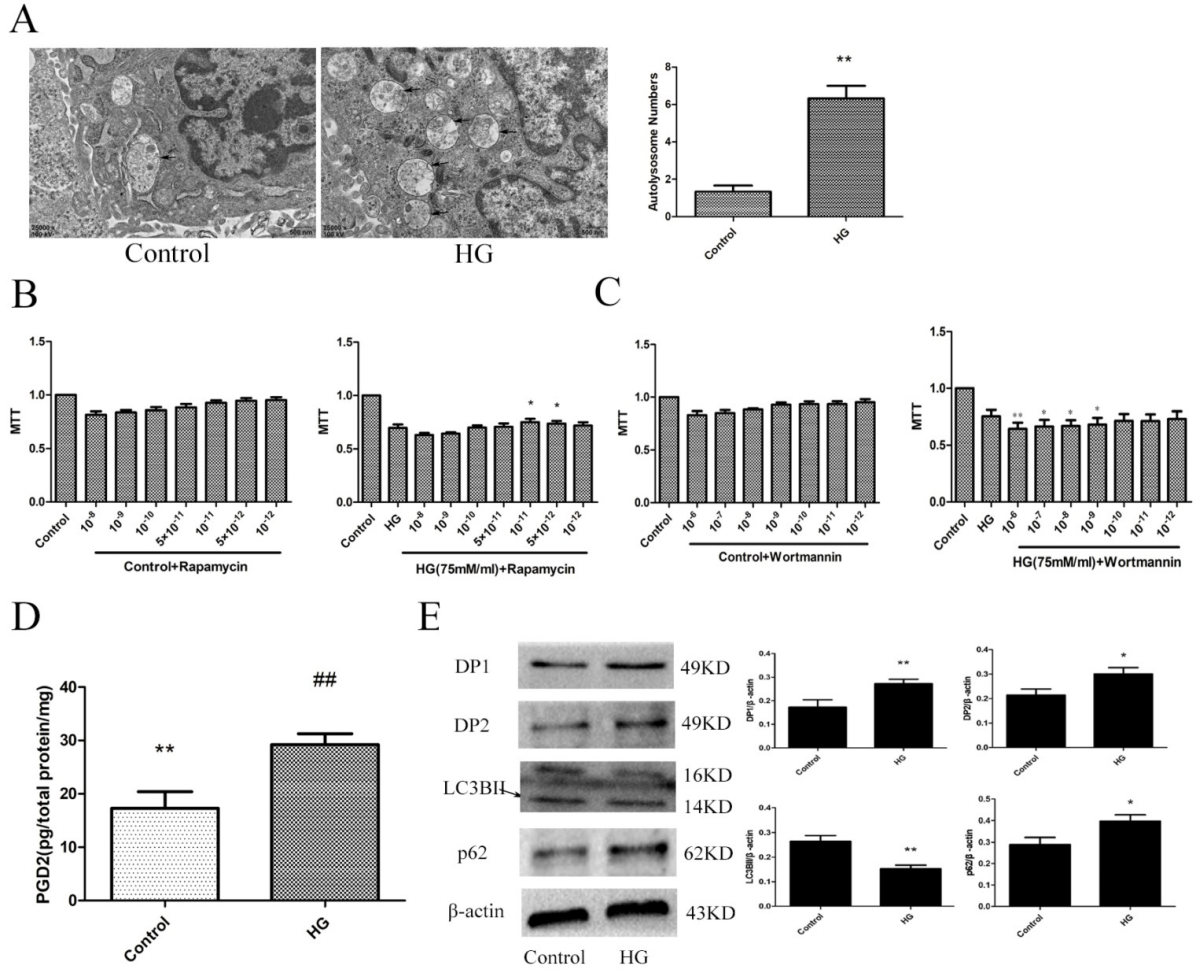
** The effect of HG on the HT22. (A)** The change of autophagolysosome was detected by electron microscopy (the arrow points to the autophagy lysosome). **(B)** The effect of autophagy agonist in HG HT22 survival. **(C)** The effect of autophagy inhibitor in HG HT22 survival. **(D)** Change of PGD2 in HG HT22. **(E)** Change of protein in HG HT22. Compare with the control, the autophagolysosome was significantly increased in HG HT22. The autophagy agonist significantly improved the HT22 injury in HG HT22, and the autophagy inhibitor significantly aggravated the HT22 injury in HG HT22. Compare with the control, the content of PGD2 was significantly increased in HG HT22. Compare with the control, the expression of DP1, DP2 and p62 was significantly increased in the HG HT22; and the expression of LC3BII was significantly decreased in the HG HT22. Data are expressed as mean ± SD of nine individual rats in each group.^*^P<0.05 and ^**^P<0.01 compared with control group, respectively.

To determine the changes in the PGD2-DP pathway and autophagy levels in HT22 cells damaged by HG, we used electron microscopy, MTT, ELISA and WB analyses to measure the related targets. An increase in autolysosomes may reflect a reduction in degradative activity 19. Combined with the increase in LC3BII expression and the decrease in p62 expression, these results indicated that the autophagy level of the HG group was decreased. Wortmannin significantly aggravated HG damage, and rapamycin significantly improved HG damage. Our results also showed that the PGD2 content, DP1 expression and DP2 expression were significantly increased in the HG group. The above findings suggested that the imbalance of PGD2-DPs may be closely related to the decrease in autophagy levels in HT22 cells damaged by HG (Figure 3).

### The effect of DP2 on the injury of HT22 caused by HG

**Figure 4:**
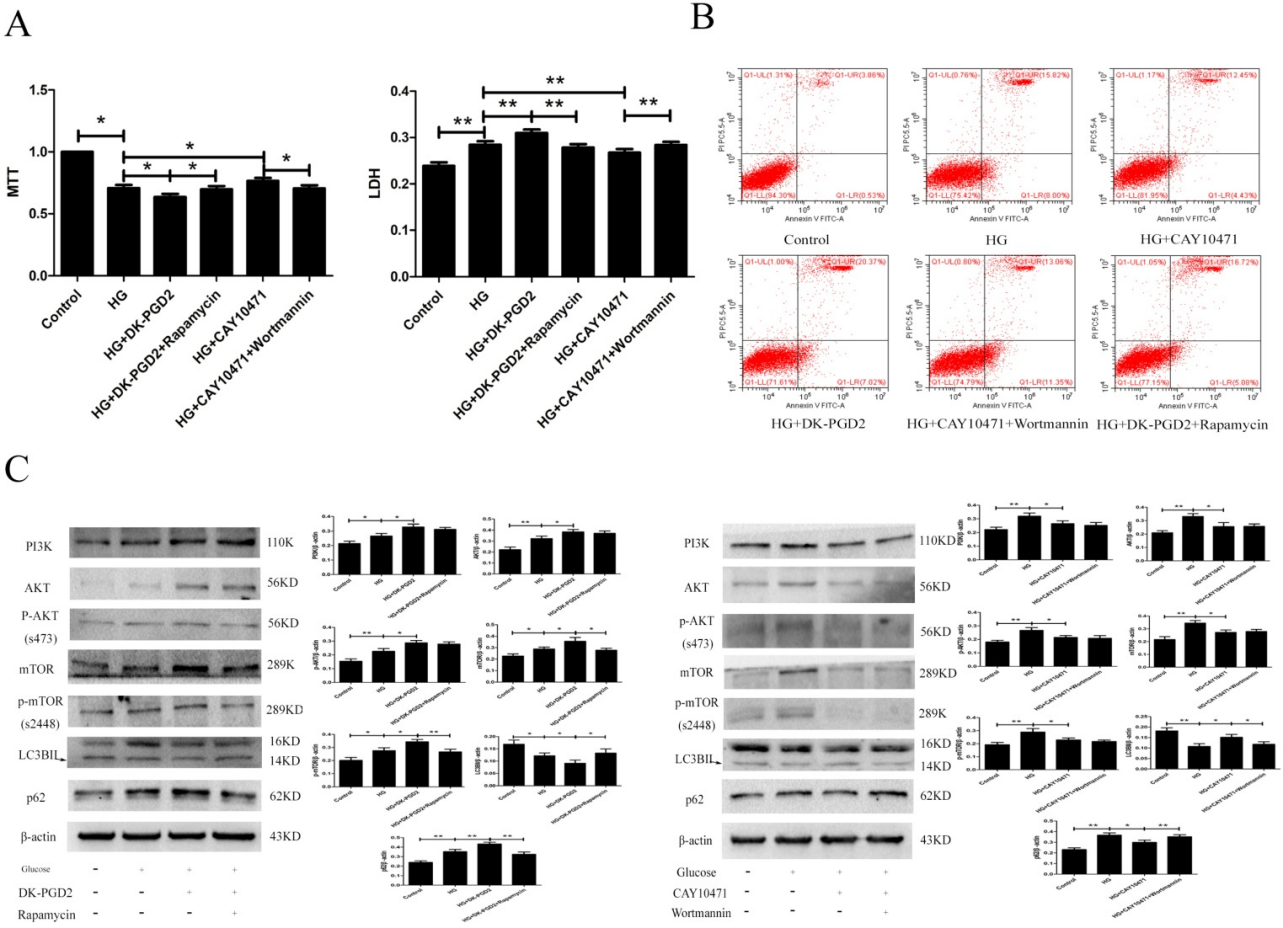
** The effect of DP2 agonistand inhibitor on the HG HT22. (A)** The effects of DP2 agonist and inhibitor on HG HT22 survival. **(B)** The effects of DP2 agonist and inhibitor on apoptosis and necrosis in HG HT22. **(C)** The effects of DP2 agonist and inhibitor on change of PI3K, AKT, p-AKT, mTOR, p-mTOR, LC3BII and p62 expression in HG HT22. Data are expressed as mean ± SD of nine individual rats in each group.^*^P<0.05 and ^**^P<0.01, respectively.

To determine the effect of DP2on HG-induced HT22 damage, we used the MTT, LDH, ELISA, flow cytometry and WB methods to measure related targets. Our results showed that the PI3K/AKT/mTOR pathway was activated in the HG group. DK-PGD2 could further activate the PI3K/AKT/mTOR pathway and downregulate autophagy to improve the HT22 damage caused by HG. CAY10471 could inhibit the PI3K/AKT/mTOR pathway and upregulate autophagy to improve the HT22 damage caused by HG. Moreover, the effects of DK-PGD2 and CAY10471 were partially reversed by rapamycin and wortmannin, respectively. The above results indicated that PGD2 inhibited autophagy through DP2-PI3K/AKT/mTOR to aggravate HT22 cell damage by HG (Figure 4).

### The effect of DP1 in HT22 injury caused by HG

**Figure 5:**
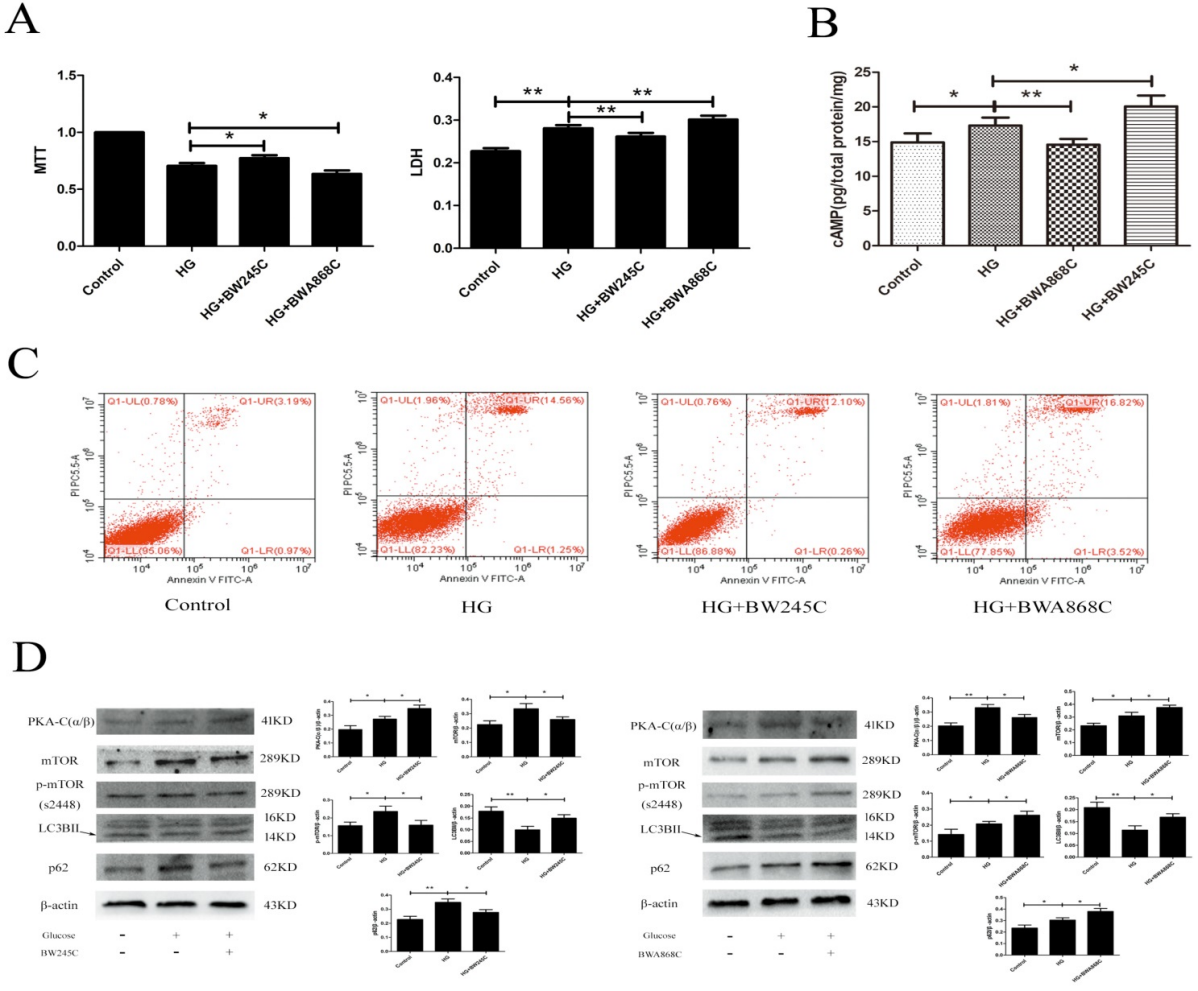
** The effect of DP1 agonistand inhibitor on the HG HT22. (A)** The effects of DP1 agonist and inhibitor on HG HT22 survival. **(B)** The effects of DP1 agonist and inhibitor on the change of cAMP in HG HT22. **(C)** The effects of DP1 agonist and inhibitor on apoptosis and necrosis in HG HT22. **(D)**The effects of DP1 agonist and inhibitor on change of PKA-C(α/β), mTOR, LC3BII and p62 expression in HG HT22. Data are expressed as mean ± SD of nine individual rats in each group.^ *^P<0.05 and ^**^P<0.01, respectively.

To determine the effect of DP1 on HG-induced HT22 damage, we used the MTT, LDH, flow cytometry and WB methods to measure the related targets. MTT measures the cell survival rate, while LDH measures the cell death rate. Our results showed that the cAMP/PKA/mTOR pathway was activated in the HG group. BW245C could further activate the cAMP/PKA/mTOR pathway and upregulate autophagy to improve HT22 damage caused by HG. However, BWA868C could further inhibit the cAMP/PKA/mTOR pathway and downregulate autophagy to aggravate the HT22 damage caused by HG. The above results indicated that PGD2 promotes autophagy through DP1-cAMP/PKA/mTOR to protect HT22 cell damage by HG (Figure 5).

### Protein interaction test

**Figure 6:**
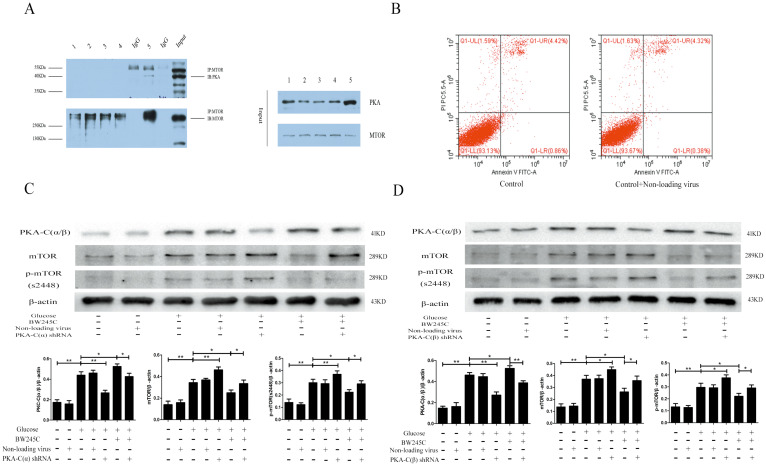
** The direct link between PKA**-**C and mTOR, and the effect of knockout PKA catalytic subunits α and β on HT22 in HG group. (A)** The interaction between PKA-C and mTOR. **(B)** The effects of non-loading virus on HG HT22. **(C)** The effect of knockout PKA catalytic subunits α and βon change of PKA-C(α/β), mTOR and p-mTOR expression in HG HT22.Data are expressed as mean ± SD of nine individual rats in each group.^ *^P<0.05 and ^**^P<0.01, respectively.

To determine whether PKA-C(α/β) and mTOR directly interact, we used a Co-IP experiment to investigate the binding between the two proteins. The Co-IP results proved that mTOR and PKA-C(α/β) directly interact in the hippocampus. We did not observe this in the cells. The reason for this result may be that cell protein levels were too low (Figure 6A). These results indicated that PKA-C(α/β) may be directly related to mTOR.

### The effect of PKA-C(α/β) shRNA on HT22 injury caused by HG

To determine whether PKA-C(α) and PKA-C(β) were involved in the regulation of mTOR, we knocked down PKA-C(α) and PKA-C(β) to explore their relationship with mTOR. Our experimental results showed that the expression of mTOR was significantly decreased in the HG group when shRNA was used to knock down PKA-C(α) and PKA-C(β). BW245C partially reversed the effect of PKA-C(α) shRNA and PKA-C(β) shRNA. The reason for the increase in PKA-C(α/β) expression was that BW245C increases the expression of another PKA subtype to decrease the expression of mTOR when PKA-C(α) or PKA-C(β) is knocked down. The above results indicated that both the α and β catalytic subunits of PKA are directly involved in the regulation of mTOR (Figure 6C,D).

## Discussion

Diabetes has become a serious social problem, and T2D accounts for the majority of the cases of diabetes. The brain damage caused by T2D has consistently received attention because T2D can significantly increase the risk of cognitive impairment 4. The reasons for T2D brain injury remain unknown.

We all know that insulin resistance is an important reason of diabetes complications. But, patients with type 2 diabetes are not necessarily insulin resistance. Some studies have found that brain had the ability to secrete insulin locally, and insulin resistance in the Central Nervous System is also considered to be directly related to neurodegeneration, which also involves autophagy inhibition 20. However, there is unequivocal evidence for selective, regulated, time dependent, temperature sensitive, carrier mediated, and saturable insulin transport to the brain 21. Some studies also have proved that insulin levels in the cerebrospinal fluid of patients with AD are significantly reduced 22. Therefore, even though the brain can secrete some insulin by itself, it is not enough to maintain normal or higher levels for long time when the insulin level was significantly decreased in the blood. Insulin resistance, the blood insulin level should be normal or elevated. However, our modeling method is to directly partially destroy part of the pancreatic islet β cells and the content of insulin is significantly decreased in blood. Insulin resistance does not exist or the time is very short, so its effect on autophagy is limited. Moreover, the duration of insulin resistance varies from person to person, some are long and some are short, and not everyone has insulin resistance. We should also note that some people do not have insulin resistance, and some people have insulin resistance for a very short time. The American Diabetes Association (ADA) “Standards of Medical Care in Diabetes” indicated that insulin resistance ultimately leads to insufficient insulin levels in T2D 23. Our animal model is more in line with the middle and late stages of T2D. Our research results may provide a new possible therapeutic target for patients who have developed T2DM in the late stages with brain damage.

In our study, we found that blood glucose, T-CHO, and TG were significantly increased; body weight and insulin were significantly decreased; and LDL-C was not significantly altered in the T2D model group. These changes are consistent with the characteristics of T2D. The Morris water maze results showed that the rats in all the groups exhibited a rapid reduction in their escape latencies to find the platform over the 4 training days. Compared with those in the control group, rats in the model group showed significantly prolonged escape latency and exhibited a decrease in the number of platform crossings at 5 days. In the control group, the morphological neuronal structure of the hippocampus and cortex was intact and clear. Compared with those of the control rats, the neurons of the T2D model rats showed significantly apoptosis in the hippocampus and cortex, and in these regions, PGD2 was significantly increased; DP2 and p62 expression was significantly increased; and DP1 and LC3BII expression was significantly decreased. We also found that the numbers of autolysosomes were significantly increased in HG cells. Many studies have indicated that greater numbers of autolysosomes are associated with lower levels of autophagy 19. In HG cells, PGD2 was significantly increased, DP1, DP2, and p62 expression was significantly increased, and LC3BII expression was significantly decreased compared with that in control cells. The expression of DP1 in the cell was significantly increased, and the expression of DP1 in the cortex and hippocampus was significantly decreased. The receptor may be desensitized and endocytosis if it is activated for a long time, resulting in a decrease in expression 24, 25. Our animal model takes up 5 months to build, while the cell model only takes 36 hours. In the animal model, PGD2 acts on the receptor for several months, while in the cell model it only acts for 36 hours. Therefore, the long-term action of PGD2 leads to the desensitization and endocytosis of the receptor and the decrease in expression. DP1 expression may be elevated to protect against acute injury. Our previous studies found that DP1 expression was significantly increased and had a protective effect on primary cultured rat hippocampal neurons injured by aluminium overload 26. We also found that autophagy inhibitors could promote HG damage, whereas autophagy agonists could reduce HG damage. These results indicated that T2D could cause brain damage and is associated with changes in PGD2-DPs and decreases in autophagy.

Recently, PGD2 has received increased research attention. The effect of PGD2 on the central nervous system is still controversial. Decreased PGD2 in depression is associated with depressive behaviour 27, but other studies have indicated that PGD2 promotes apoptosis in APP/PS1 mice 28. The controversy may stem from the effects and expression of PGD2's corresponding receptors, DP1 and DP2. The main effect of DP2 is to cause damage, including hypoxic-ischaemic brain damage 29 and AD 28. However, the effect of DP1 is different. DP1 knockdown has an obvious protective effect on intracerebral haemorrhage 30 and can significantly aggravate cerebral ischaemia-reperfusion 31. Therefore, the effect of DP1 and DP2 needs to be verified.

Our results showed that the DP2 agonist DK-PGD2 significantly decreased cell survival and increased cell death, necrosis, and apoptosis; the DP2 inhibitor CAY10471 significantly increased cell survival and decreased cell death, necrosis, and apoptosis in the HG group. These results suggested that DP2 promotes brain damage in T2D. Similar results have been found in other studies.DP2 inhibition can play a significant neuroprotective role in PD 32, andDP2 mediates depression-like behaviour in a mouse model of depression 33.

DP2 is a G-protein-coupled receptor and can activate cGMP/PKC and PI3K/AKT 34-36. DP2 involves the activation of multiple neuronal damage pathways, and the classic autophagy inhibitory PI3K/AKT/mTOR pathway may be one of them.To confirm that DP2 is involved in T2D brain injury by activating PI3K/AKT/mTOR to inhibit autophagy, rapamycin and wortmannin were administered to DK-PGD2 and CAY10471, respectively. Our results showed that the PI3K/AKT/mTOR pathway was activated in the HG group. DK-PGD2 could further activatethe PI3K/AKT/mTOR pathway and downregulate autophagy to improve HT22 damage caused by HG. CAY10471 could inhibit the PI3K/AKT/mTOR pathway and upregulate autophagy to improve the HT22 damage caused by HG. Moreover, the effects of DK-PGD2 and CAY10471 were partially reversed by rapamycin and wortmannin, respectively. The above results indicated that PGD2 inhibited autophagy through DP2-PI3K/AKT/mTOR to aggravate the HT22 cell damage by HG.

DP1 is another receptor for PGD2. In the HG group, the DP1 agonist BW245C significantly increased cell survival and decreased cell death, necrosis, and apoptosis; the DP1 inhibitor BWA868C significantly decreased cell survival and increased cell death, necrosis, and apoptosis. These results suggest that DP1 protects against brain damage in T2D. Some studies had similar results 31; for example, one group found that DP1 gene knockout could significantly aggravate cerebral ischaemia-reperfusion injury. However, some studies 34 produced results different from ours; for example, one study found that DP1 gene knockout had an obvious protective effect against cerebral haemorrhage. These results suggest that the effect of DP1 is different in different models.

DP1 is also a G-protein-coupled receptor and can increase cAMP. cAMP is a key factor in the activation of PKA 37 and can release PKA-Cs to exert effects by binding to the PKA regulatory subunit (PKA-R) 38. Some researchers believe that PKA has a regulatory effect on autophagy 39, and PKA-regulated autophagy may be related to mTOR 40. BW245C could further activate the cAMP/PKA/mTOR pathway and upregulate autophagy to improve HT22 damage caused by HG. However, BWA868C could further inhibit the cAMP/PKA/mTOR pathway and downregulate autophagy to aggravate the HT22 damage caused by HG. The above results indicated that PGD2 promotes autophagy through DP1-cAMP/PKA/mTOR to protect against the HT22 celldamage by HG.

The above studies do not show whether PKA-C(α/β) and mTOR have a direct relationship. Therefore, we used Co-IP experiments to determine whether mTOR and PKA-C interact directly. Our Co-IP results suggested that PKA-C(α/β) and mTOR interact directly. There are three subtypes of PKA-C:α is widely distributed in all tissues, β is highly expressed in brain tissue, and γis only expressed in testicular tissue 41-43. The above experiment did not indicate which subtype decreases mTOR expression. Therefore, we used shRNA to silence PKA-C(α) and PKA-C(β). Our results showed that the expression of mTOR was significantly increased when PKA-C(α) orPKA-C(β) was silenced. The effect of BW245C on increasing PKA-C(α/β) was also significantly attenuated when PKA-C(α)or PKA-C(β)was silenced, and the expression of mTOR and p-mTOR (s2448) was significantly increased. Some studies 44 have also found that PKA not only decreases the expression of mTOR and p-mTOR (S2448) but also decreases the expression of p-mTOR (S2481). The above studies combined with our research indicate that mTOR could be degraded by PKA as its substrate. The significant decrease in mTOR expression may be related to the ubiquitination of mTOR. Studies have found that mTOR can be degraded by ubiquitin 45. PKA-C promotes the ubiquitin-mediated degradation of NLRP3 by phosphorylating its s291 site 46. Therefore, we hypothesize that PKA phosphorylates a site in mTOR, which leads to the enhancement of mTOR ubiquitination and accelerates mTOR degradation, but subsequent experiments are needed to prove this hypothesis.

In the HG group, we found that the content of cAMP and the expression of mTOR and p-mTOR (s2448) were significantly increased. However, compared with the HG group, BW245C significantly increased the content of cAMP and significantly decreased the expression of mTOR and p-mTOR (s2448). Some studies have indicated that PGE2 and cAMP increase mTOR activation in some experimental systems 47, 48; the suppressive actions observed in our system are consistent with other reports of mTOR inhibition by cAMP 49 and by PKA 50. The reason for this phenomenon may be that the cAMP increase is not enough to activate sufficient PKA to decrease mTOR; therefore, the increase in cAMP decreases mTOR when DP1 is stimulated. PKA-R hasfour subtypes, RIα, RIβ, RIIα, and RIIβ. Some studies have indicated that PKA containing RIα or RIβ could be activated at low levels of cAMP, but PKA containing RIIα or RIIβ requires higher levels of cAMP for activation 51. Our results showed that the expression of mTOR was significantly increased when PKA-C(α) or PKA-C(β) was silenced in the HG group. This result indicated that PKA also had this effect on the cAMP concentration of the HG group, but this concentration of cAMP was not enough to activate PKA containing RIIα or RIIβ. Therefore, the expression of mTOR was significantly decreased when BW245C continued to increase the cAMP concentration and activate more PKA.

Our results indicate that the imbalance of PGD2-DPs might be related to changes in autophagy levels, and PGD2 is involved in T2D brain damage by regulating autophagy via the DP1-PKA/mTOR and DP2-PI3K/AKT/mTOR pathways. PGD2 may promote autophagy through DP1-PKA/mTOR to protect against T2D brain damage and may inhibit autophagy through DP2-PI3K/AKT/mTOR to aggravate T2D brain damage. Therefore, DP1 stimulation or DP2 inhibition can be used as therapeutic targets for T2D brain injury.

## Author Contributions

Junqing Yang made substantial contribution to conception and design andperformance of the study. Yang Yang, Pu Xiang, Qi Chen, Ying Luo, Hong Wang, Huan Li, Lu Yang, Congli Hu, Jiahua Zhang, Yuke Li, Hui Xia and Zhihao Chenparticipated in performance of all experiments and carried out the data analysis. Yang Yang participated in performance of the study and inwriting the manuscript. All authors read and approved the final manuscript.

## Figures and Tables

**Figure 7:**
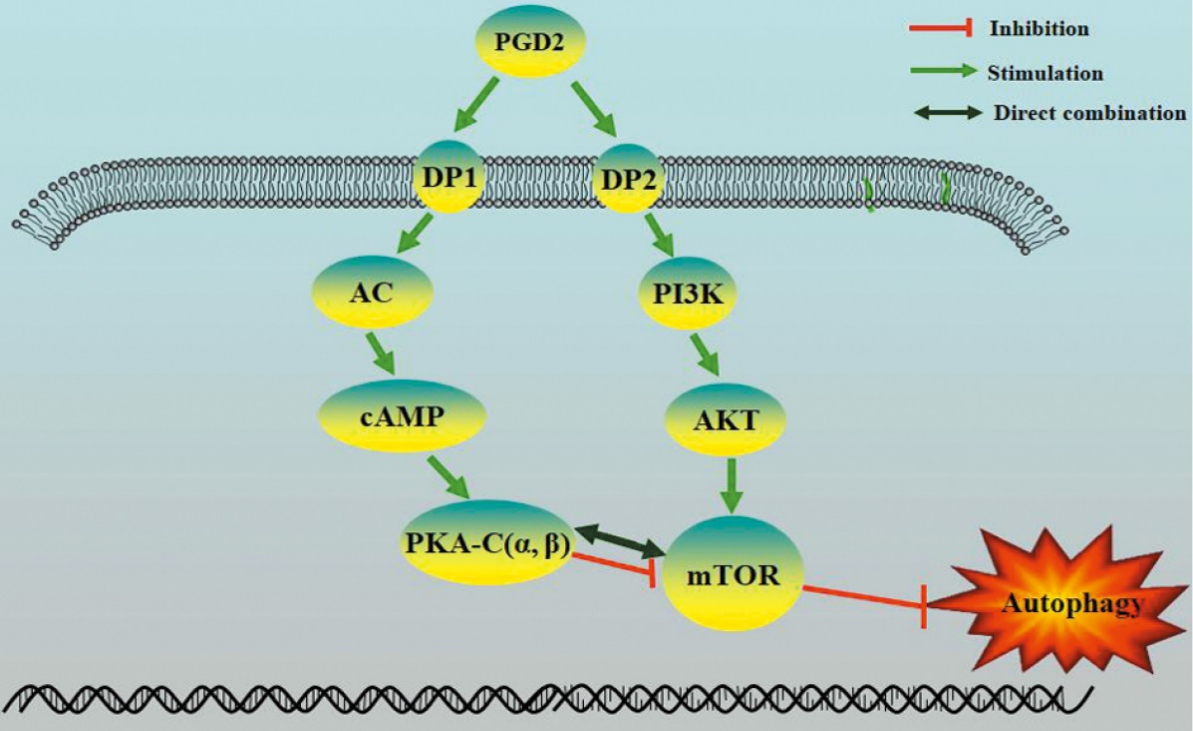
Proposed model of regulation of autophagy by PGD2 in neurons.

## References

[1] Sima AA (2010). Encephalopathies: the emerging diabetic complications. Acta Diabetol.

[2] Sebastiao I, Candeias E, Santos MS, de Oliveira CR, Moreira PI, Duarte AI (2014). Insulin as a Bridge between Type 2 Diabetes and Alzheimer Disease - How Anti-Diabetics Could be a Solution for Dementia. Front Endocrinol (Lausanne).

[3] Zimmet P (2009). Preventing diabetic complications: a primary care perspective. Diabetes Res Clin Pract.

[4] Sharma G, Parihar A, Talaiya T, Dubey K, Porwal B, Parihar MS (2020). Cognitive impairments in type 2 diabetes, risk factors and preventive strategies. J Basic Clin Physiol Pharmacol.

[5] Tavernarakis N (2020). Regulation and Roles of Autophagy in the Brain. Adv Exp Med Biol.

[6] Ling D, Salvaterra PM (2011). Brain aging and Abeta(1)(-)(4)(2) neurotoxicity converge via deterioration in autophagy-lysosomal system: a conditional Drosophila model linking Alzheimer's neurodegeneration with aging. Acta Neuropathol.

[7] Luo R, Su LY, Li G, Yang J, Liu Q, Yang LX (2020). Activation of PPARA-mediated autophagy reduces Alzheimer disease-like pathology and cognitive decline in a murine model. Autophagy.

[8] Zhang J, Zhang JX, Zhang QL (2016). PI3K/AKT/mTOR-mediated autophagy in the development of autism spectrum disorder. Brain Res Bull.

[9] Candeias E, Sebastiao I, Cardoso S, Carvalho C, Santos MS, Oliveira CR (2018). Brain GLP-1/IGF-1 Signaling and Autophagy Mediate Exendin-4 Protection Against Apoptosis in Type 2 Diabetic Rats. Mol Neurobiol.

[10] Kobayashi S, Xu X, Chen K, Liang Q (2012). Suppression of autophagy is protective in high glucose-induced cardiomyocyte injury. Autophagy.

[11] Yagami T, Koma H, Yamamoto Y (2016). Pathophysiological Roles of Cyclooxygenases and Prostaglandins in the Central Nervous System. Mol Neurobiol.

[12] Ahmad AS, Ottallah H, Maciel CB, Strickland M, Dore S (2019). Role of the L-PGDS-PGD2-DP1 receptor axis in sleep regulation and neurologic outcomes. Sleep.

[13] Mesarwi O, Polak J, Jun J, Polotsky VY (2013). Sleep disorders and the development of insulin resistance and obesity. Endocrinol Metab Clin North Am.

[14] Lai YJ, Lin CL, Lin MC, Lee ST, Sung FC, Chang YJ (2013). Population-based cohort study on the increase in the risk for type 2 diabetes mellitus development from nonapnea sleep disorders. Sleep Med.

[15] Yang Y, Chen Q, Zhao Q, Luo Y, Xu Y, Du W (2019). Inhibition of COX2/PGD2-Related Autophagy Is Involved in the Mechanism of Brain Injury in T2DM Rat. Front Cell Neurosci.

[16] Ma J, Li H, Hu X, Yang L, Chen Q, Hu C (2017). CMD-05, a novel promising clinical anti-diabetic drug candidate, *in vivo* and vitro studies. Sci Rep.

[17] Li Y, Chen Q, Ran D, Wang H, Du W, Luo Y (2019). Changes in the levels of 12/15-lipoxygenase, apoptosis-related proteins and inflammatory factors in the cortex of diabetic rats and the neuroprotection of baicalein. Free Radic Biol Med.

[18] Yang L, Han W, Luo Y, Hu X, Xu Y, Li H (2018). Adapentpronitrile, a New Dipeptidyl Peptidase-IV Inhibitor, Ameliorates Diabetic Neuronal Injury Through Inhibiting Mitochondria-Related Oxidative Stress and Apoptosis. Front Cell Neurosci.

[19] Klionsky DJ, Abdelmohsen K, Abe A, Abedin MJ, Abeliovich H, Acevedo Arozena A (2016). Guidelines for the use and interpretation of assays for monitoring autophagy (3rd edition). Autophagy.

[20] de Mello NP, Orellana AM, Mazucanti CH, de Morais Lima G, Scavone C, Kawamoto EM (2019). Insulin and Autophagy in Neurodegeneration. Front Neurosci.

[21] Akintola AA, van Heemst D (2015). Insulin, aging, and the brain: mechanisms and implications. Front Endocrinol (Lausanne).

[22] Craft S, Peskind E, Schwartz MW, Schellenberg GD, Raskind M, Porte D Jr (1998). Cerebrospinal fluid and plasma insulin levels in Alzheimer's disease: relationship to severity of dementia and apolipoprotein E genotype. Neurology.

[23] American Diabetes A (2019). 2. Classification and Diagnosis of Diabetes: Standards of Medical Care in Diabetes-2019. Diabetes Care.

[24] Milatovic D, Montine TJ, Aschner M (2011). Prostanoid signaling: dual role for prostaglandin E2 in neurotoxicity. Neurotoxicology.

[25] Kelly E, Bailey CP, Henderson G (2008). Agonist-selective mechanisms of GPCR desensitization. Br J Pharmacol.

[26] Ma J, Yang Q, Wei Y, Yang Y, Ji C, Hu X (2016). Effect of the PGD2-DP signaling pathway on primary cultured rat hippocampal neuron injury caused by aluminum overload. Sci Rep.

[27] Chu C, Wei H, Zhu W, Shen Y, Xu Q (2017). Decreased Prostaglandin D2 Levels in Major Depressive Disorder Are Associated with Depression-Like Behaviors. Int J Neuropsychopharmacol.

[28] Guo JW, Guan PP, Ding WY, Wang SL, Huang XS, Wang ZY (2017). Erythrocyte membrane-encapsulated celecoxib improves the cognitive decline of Alzheimer's disease by concurrently inducing neurogenesis and reducing apoptosis in APP/PS1 transgenic mice. Biomaterials.

[29] Sun LQ, Guo GL, Zhang S, Yang LL (2018). Effects of MicroRNA-592-5p on Hippocampal Neuron Injury Following Hypoxic-Ischemic Brain Damage in Neonatal Mice - Involvement of PGD2/DP and PTGDR. Cell Physiol Biochem.

[30] Ahmad AS, Mendes M, Hernandez D, Dore S (2017). Efficacy of Laropiprant in Minimizing Brain Injury Following Experimental Intracerebral Hemorrhage. Sci Rep.

[31] Saleem S, Zhuang H, de Brum-Fernandes AJ, Maruyama T, Narumiya S, Dore S (2007). PGD(2) DP1 receptor protects brain from ischemia-reperfusion injury. Eur J Neurosci.

[32] Corwin C, Nikolopoulou A, Pan AL, Nunez-Santos M, Vallabhajosula S, Serrano P (2018). Prostaglandin D2/J2 signaling pathway in a rat model of neuroinflammation displaying progressive parkinsonian-like pathology: potential novel therapeutic targets. J Neuroinflammation.

[33] Onaka Y, Shintani N, Nakazawa T, Haba R, Ago Y, Wang H (2015). CRTH2, a prostaglandin D2 receptor, mediates depression-related behavior in mice. Behav Brain Res.

[34] Hirai H, Tanaka K, Takano S, Ichimasa M, Nakamura M, Nagata K (2002). Cutting edge: agonistic effect of indomethacin on a prostaglandin D2 receptor, CRTH2. J Immunol.

[35] Takeshita K, Yamasaki T, Nagao K, Sugimoto H, Shichijo M, Gantner F (2004). CRTH2 is a prominent effector in contact hypersensitivity-induced neutrophil inflammation. Int Immunol.

[36] Hata AN, Breyer RM (2004). Pharmacology and signaling of prostaglandin receptors: multiple roles in inflammation and immune modulation. Pharmacol Ther.

[37] Hirata M, Kakizuka A, Aizawa M, Ushikubi F, Narumiya S (1994). Molecular characterization of a mouse prostaglandin D receptor and functional expression of the cloned gene. Proc Natl Acad Sci U S A.

[38] Corbin JD, Sugden PH, West L, Flockhart DA, Lincoln TM, McCarthy D (1978). Studies on the properties and mode of action of the purified regulatory subunit of bovine heart adenosine 3':5'-monophosphate-dependent protein kinase. J Biol Chem.

[39] Torres-Quiroz F, Filteau M, Landry CR (2015). Feedback regulation between autophagy and PKA. Autophagy.

[40] Wu J, Gao F, Xu T, Deng X, Wang C, Yang X (2018). miR-503 suppresses the proliferation and metastasis of esophageal squamous cell carcinoma by triggering autophagy via PKA/mTOR signaling. Int J Oncol.

[41] Cadd G, McKnight GS (1989). Distinct patterns of cAMP-dependent protein kinase gene expression in mouse brain. Neuron.

[42] Foss KB, Simard J, Berube D, Beebe SJ, Sandberg M, Grzeschik KH (1992). Localization of the catalytic subunit C gamma of the cAMP-dependent protein kinase gene (PRKACG) to human chromosome region 9q13. Cytogenet Cell Genet.

[43] Chang A, Li PP, Warsh JJ (2003). Altered cAMP-dependent protein kinase subunit immunolabeling in post-mortem brain from patients with bipolar affective disorder. J Neurochem.

[44] Arnaud-Batista FJ, Peruchetti DB, Abreu TP, do Nascimento NR, Malnic G, Fonteles MC (2016). Uroguanylin modulates (Na++K+)ATPase in a proximal tubule cell line: Interactions among the cGMP/protein kinase G, cAMP/protein kinase A, and mTOR pathways. Biochim Biophys Acta.

[45] Wang FF, Zhang XJ, Yan YR, Zhu XH, Yu J, Ding Y (2017). FBX8 is a metastasis suppressor downstream of miR-223 and targeting mTOR for degradation in colorectal carcinoma. Cancer Lett.

[46] Guo C, Xie S, Chi Z, Zhang J, Liu Y, Zhang L (2016). Bile Acids Control Inflammation and Metabolic Disorder through Inhibition of NLRP3 Inflammasome. Immunity.

[47] Misra UK, Pizzo SV (2009). Epac1-induced cellular proliferation in prostate cancer cells is mediated by B-Raf/ERK and mTOR signaling cascades. J Cell Biochem.

[48] Kim HW, Ha SH, Lee MN, Huston E, Kim DH, Jang SK (2010). Cyclic AMP controls mTOR through regulation of the dynamic interaction between Rheb and phosphodiesterase 4D. Mol Cell Biol.

[49] Okunishi K, DeGraaf AJ, Zaslona Z, Peters-Golden M (2014). Inhibition of protein translation as a novel mechanism for prostaglandin E2 regulation of cell functions. FASEB J.

[50] Xie J, Ponuwei GA, Moore CE, Willars GB, Tee AR, Herbert TP (2011). cAMP inhibits mammalian target of rapamycin complex-1 and -2 (mTORC1 and 2) by promoting complex dissociation and inhibiting mTOR kinase activity. Cell Signal.

[51] Yang H, Yang L (2016). Targeting cAMP/PKA pathway for glycemic control and type 2 diabetes therapy. J Mol Endocrinol.

